# Quantum Mechanics/Fluctuating Charge Protocol to Compute Solvatochromic Shifts

**DOI:** 10.1021/acs.jctc.1c00763

**Published:** 2021-10-07

**Authors:** Matteo Ambrosetti, Sulejman Skoko, Tommaso Giovannini, Chiara Cappelli

**Affiliations:** Scuola Normale Superiore, Piazza dei Cavalieri 7, 56126 Pisa, Italy

## Abstract

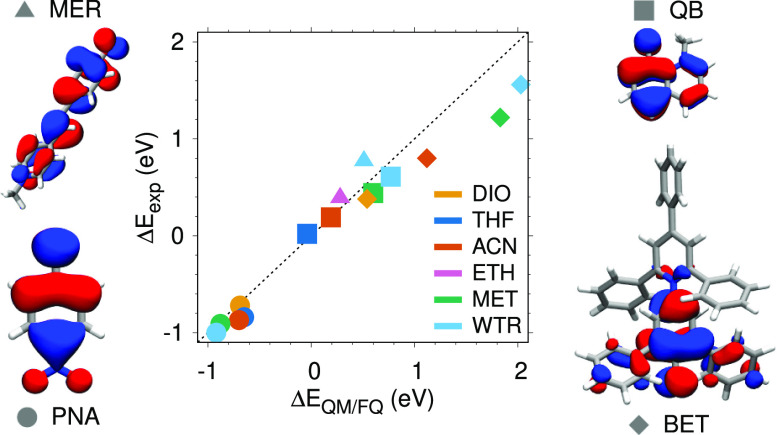

Despite the potentialities of the quantum mechanics (QM)/fluctuating charge (FQ) approach to model the spectral properties of solvated systems, its extensive use has been hampered by the lack of reliable parametrizations of solvents other than water. In this paper, we substantially extend the applicability of QM/FQ to solvating environments of different polarities and hydrogen-bonding capabilities. The reliability and robustness of the approach are demonstrated by challenging the model to simulate solvatochromic shifts of four organic chromophores, which display large shifts when dissolved in apolar, aprotic or polar, protic solvents.

## 1 Introduction

The study of electronic and optical properties of chromophores is of particular interest for many different applications, ranging from photochemistry to technology.^1^ In most cases, such optical properties are tuned by dissolving the selected dye in different solvents, which can yield to substantial changes in the solute’s properties.^2^ When dealing with electronic excitations, solvent effects mainly manifest in a shift of the solute’s absorption band,^3^ which is usually referred to as solvatochromism, or a solvatochromic shift.^2,4−10^ Depending on the nature of the transition, blue or red shifts are observed;^2^ therefore, reliable computational approaches are needed to correctly reproduce both the “sign” of the solvatochromic shift and its magnitude as a function of the nature of the solvent.^9^

To this end, different methods have been proposed, generally focusing the attention on the solute, which is responsible for the spectral signal and is accurately described at the quantum-mechanics (QM) level. The solvent, which modifies but does not determine the spectral properties, is instead treated at a lower level of sophistication.^11,12^ Various approaches differ in the way they describe the solvent, which can be treated implicitly, as a continuum, or atomistically.^12,13^ In the latter case, QM/molecular mechanics (MM)^14,15^ or quantum embedding approaches^16,17^ may be used, where solvent molecules are described by a classical force field or a QM wavefunction, respectively. Another remarkable difference between the three aforementioned approaches is the way they model solute–solvent interactions. Continuum solvation is a “mean-field” approach, where specific, directional interactions (e.g., hydrogen bonding) are neglected.^11^ The latter are instead considered in explicit, atomistic approaches, which are specified by the quality of the description of solvent molecules and their interaction with the QM solute.^14,18−21^ In most quantum embedding methods, electrostatic, polarization, and Pauli repulsion solute–solvent interactions are accurately treated,^19,22^ whereas QM/MM approaches generally discard quantum repulsion,^23,24^ and sometimes also polarization effects.^25^ However, the computational cost of QM/MM methods being much lower than those of most quantum embedding methods, the former are rapidly becoming the golden standard for many applications,^18,25,26^ especially those refined approaches, which are able to correctly take into account solute–solvent mutual polarization effects (i.e., in the so-called polarizable QM/MM embedding methods).^3,18,26−29^

Polarizable QM/MM embedding approaches can be based on distributed multipoles,^30^ induced dipoles,^3,25,27,29^ Drude oscillators,^31,32^ fluctuating charges (FQ),^33,34^ and possibly dipoles,^35−37^ or Amoeba.^38−40^ In particular, the QM/FQ approach has been specifically developed to model spectral properties.^18^ There, MM atoms are endowed with a charge that can vary as a function of differences in MM electronegativity or as a response to the electric potential generated by the QM density.^33^

**Figure 1:**
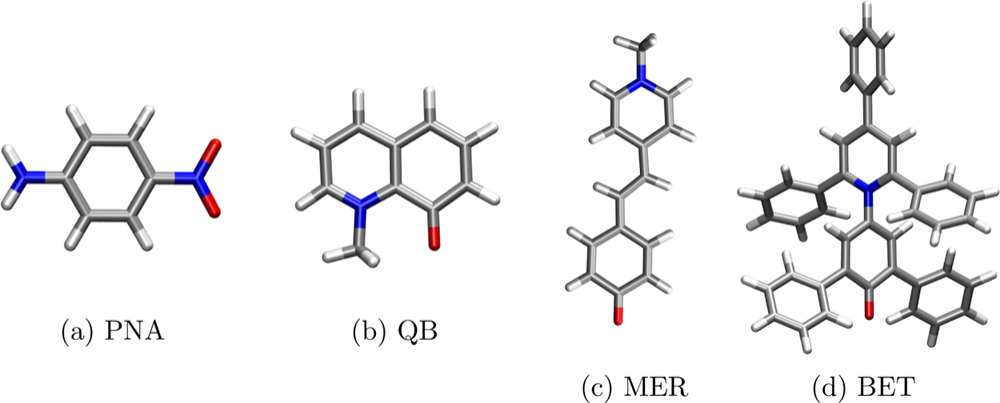
Molecular structures of the studied dyes.

Despite the excellent performance of QM/FQ to describe aqueous solutions, mainly due to a reliable parametrization of the force field and the extension up to analytical energy third derivatives,^41−47^ its application to non-aqueous solutions has been severely hampered by the lack of reliable parametrizations. In fact, QM/FQ is a completely general approach, which can be applied to any embedded system, pending a reliable parametrization of the classical layer.^18^ In this work, we extend for the first time the FQ force field to six solvents of different polarities and hydrogen-bonding capabilities, thus substantially increasing the applicability of QM/FQ beyond aqueous systems. The method is tested to reproduce solvatochromic shifts of four chromophores, which exhibit large solvatochromic shifts when dissolved in polar, protic and apolar, aprotic solvents, namely, *para*-nitroaniline (PNA), 1-methyl-8-oxyquinolinium betaine (QB), 1-methyl-4-[(oxocyclohexadienylidene)ethylidene]-1,4-dihydropyridine (MER), and 2,6-diphenyl-4-(2,4,6-triphenylpyridin-1-ium-1-yl)phenolate (BET). The molecular structures of the dyes are shown in Figure 1.

The paper is organized as follows. The next section reports on the computational methods, which have been used to parametrize the FQ force field for six selected solvents. The novel FQ parameters are then exploited to predict solvatochromic shifts of the four chromophores in the selected solvents. A summary and a discussion on the future perspectives of the method end the paper.

## 2 Methods

**Figure 2:**
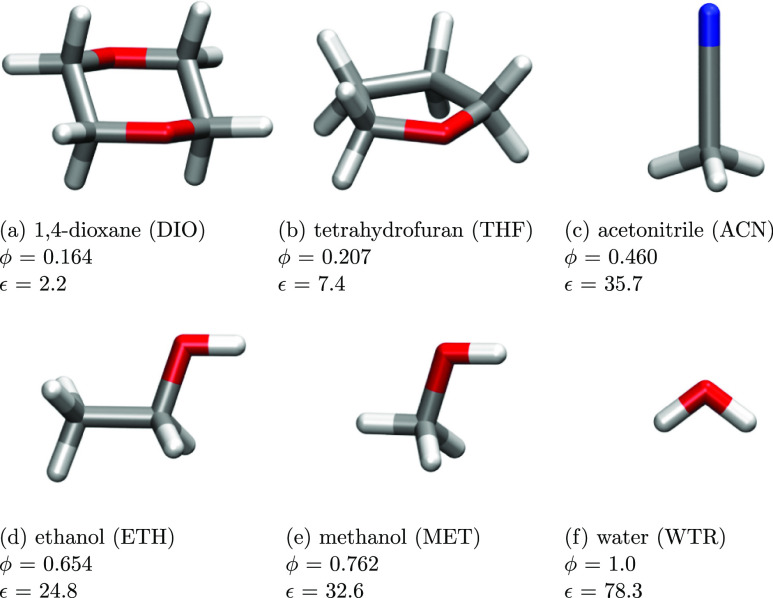
Geometries of the solvents studied in the present work ordered based on their polarity. Relative polarity (ϕ) as reported in ref (2) and the dielectric constant (ϵ) used in QM/PCM calculations are also given.

In QM/FQ, each solvent atom is endowed with a FQ, whose value can vary as a response to the QM electronic density. FQs are defined by solving a linear equation, which is written in terms of atomic electronegativities (χ) and chemical hardnesses (η), which constitute the parameters of the FQ force field. In this paper, we propose a novel parametrization for 1,4-dioxane (DIO), tetrahydrofuran (THF), acetonitrile (ACN), ethanol (ETH), methanol (MET), and water (WTR), see Figure 2, where also polarity (ϕ) and dielectric constant (ϵ) of each solvent values are reported. Notice that ϕ is equivalent to the normalized *E*_N_^T^ value usually exploited in Reichardt’s scale.^9^

### 2.1 Parameterization Procedure

**Figure 3:**
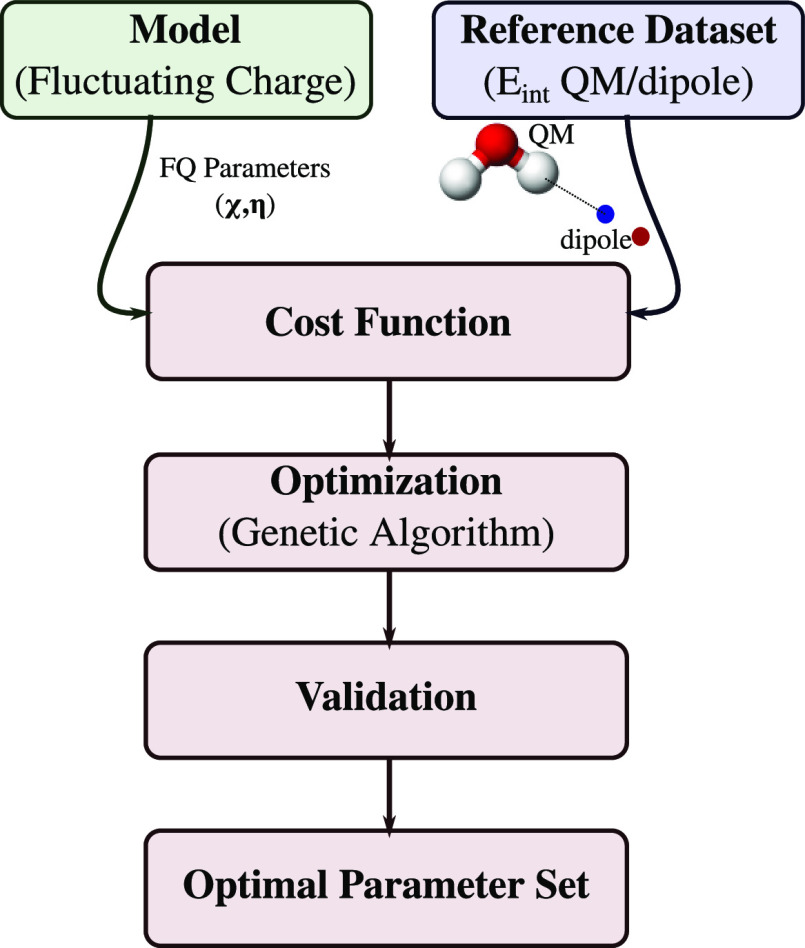
Graphical representation of the parameterization workflow.

As already mentioned above, the application of QM/FQ to different environments needs appropriate specification of atomic electronegativities and chemical hardnesses.^48−50^ In this work, we have decided to exploit atomic parameters independently of chemical makeups. For instance, in MET, the two hydrogen atoms in C–H and O–H groups are characterized by the same atomic parameters. Notice, however, that our procedure is general and specific atom types, defined similar to other force fields, can also be considered. The parameterization workflow is sketched in Figure 3.

#### 2.1.1 Reference Dataset

The first step of the parametrization procedure involves the definition of a reliable reference data set, which we built for the following four solvents of increasing polarity:^2^ DIO, ACN, MET, and WTR. Notice that, although the parameterization procedure is solvent specific, the final parameters can be transferred to similar solvents, that is, those that are constituted of similar geometrical structures or have similar physicochemical properties. This is indeed the case of THF and ETH, for which the parameters were specified by transferring those obtained for DIO and MET, respectively (vide infra). The reference data set was assembled by using solvent geometries optimized at the CCSD/aug-cc-pVTZ level of theory.

**Figure 4:**
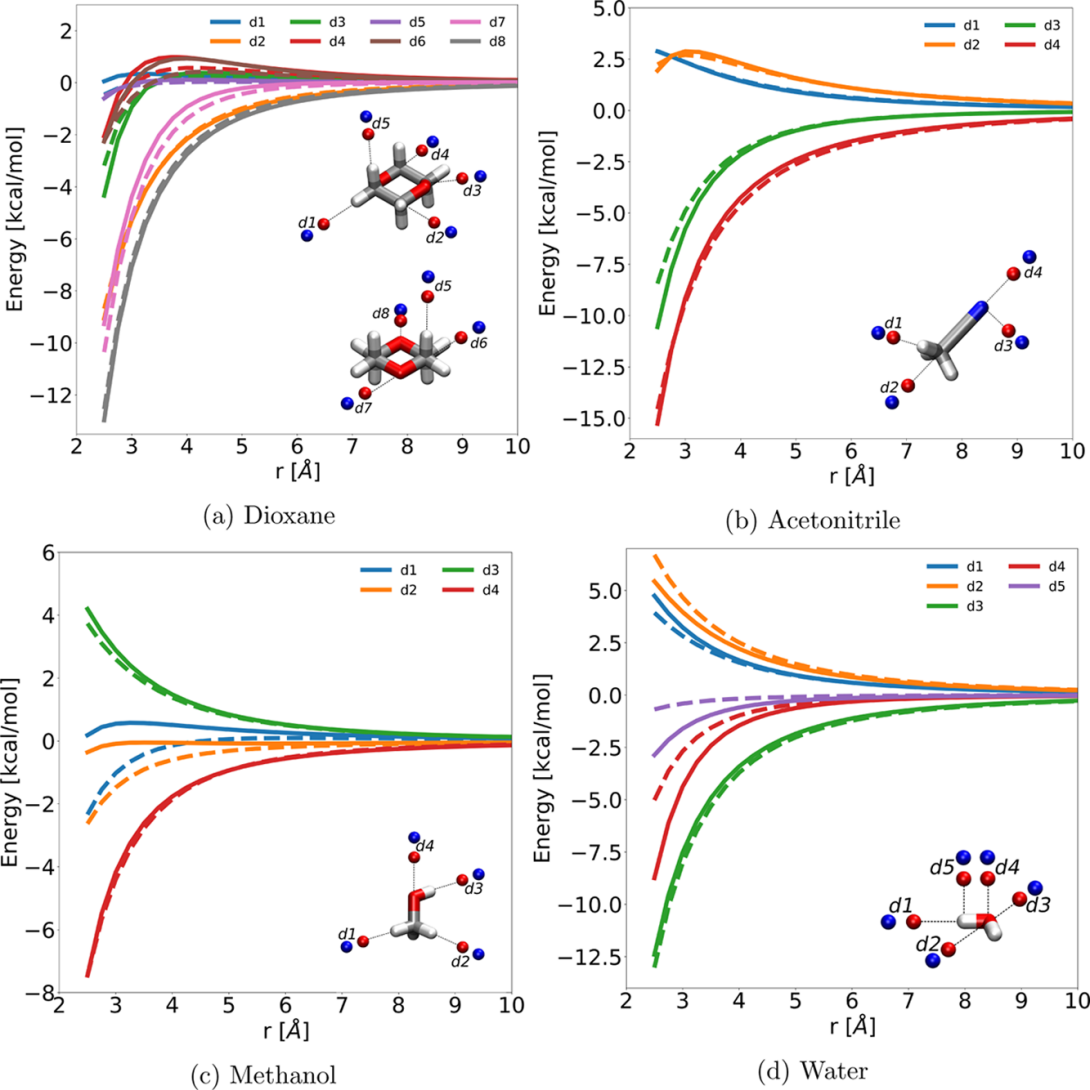
Reference QM/dipole (solid lines) and FQ/dipole (dashed lines) interaction energies (in kcal/mol) as a function of the solvent–dipole distance for all the parametrized solvents (DIO, ACN, MET, and WTR).

For all structures in the data set, the interaction energy (*E*_int_^REF^) between each solvent molecule (treated at the QM level) and a dipolar probe was calculated. All QM/dipole reference calculations were performed at the Hartree–Fock/6-311++G** level, by using the electronic structure program .^51^ In particular, we followed the strategy first proposed by Stern et al.^52^ and recently exploited in ref (53). In this approach, the dipolar probe is constituted by a pair of ±1 a.u. point charges separated by 1 Å. The solvent–dipole interaction energy is then sampled along different symmetry axes and solvent–dipole distances. Depending on the solvent, a different number of points, ranging between 150 and 250, were exploited. The minimum/maximum distance between the probe and the solvent molecule is 2.5/10.0 Å, with a constant step of 0.25 Å. The results of the calculations for the reference data set obtained for the four solvents are plotted as a solid line in Figure 4.

#### 2.1.2 Cost Function

The loss function (ξ^2^) used to train the model was defined as
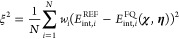
1It is a weighted mean squared error loss function, where *N* represents the number of points used in the fitting, that is, the solvent–dipole scan configurations obtained at the previous step. *E*_int,*i*_^REF^ is the QM/dipole reference interaction energy of the *i*-th geometry. On the same geometry, the FQ–dipole interaction energy (*E*_int,*i*_^FQ^(**χ**,**η**)) is computed by treating the solvent molecule at the FQ level. The FQ energy depends on **χ** and **η**, which are the adjustable parameters of the optimization. In eq 1, *w*_*i*_ is a weighting factor, which was set to the inverse of the solvent–dipole distance.

#### 2.1.3 Optimization

FQ parametrization is a non-convex optimization problem, because the two parameter sets **χ** and **η** are not linearly related to the interaction energy *E*_int,*i*_^FQ^(**χ**,**η**). As a consequence, the function is characterized by several local minima, thus a gradient-based algorithm (e.g., steepest descent, conjugate gradient method) would not be effective. For this reason, we exploited a genetic algorithm (GA), which is an evolutionary algorithm that mimics the process of natural selection.^54,55^ In particular, we used the Python module inspyred,^56,57^ where the parameter space ({**χ**, **η**}) was restrained to the [0,1] interval.

For each solvent molecule, a minimum of 20 parameter optimizations were performed, each constituted by a population of 100 individuals, corresponding to a randomly generated parameter set (**χ**, **η**). Therefore, for each molecule, 2000 different starting points in the parameter space were considered.

#### 2.1.4 Validation

GA is a stochastic optimization technique. At the end of the previous step, multiple sets of suboptimal parameters, that is, characterized by similar values of the loss function ξ^2^, were obtained. In order to select the best parameter set, we performed an additional validation step, which is based on two physical observations.

First, the parameter sets have to follow the electronegativity scale, that is, atomic electronegativity needs to increase as moving right along a row of the periodic table. Therefore, all suboptimal parameter sets that do not follow this criterion were rejected.

Second, we imposed the parameter sets to correctly reproduce the molecular static isotropic polarizability α_mol_, computed at the CCSD/aug-cc-pVTZ level, and the static isotropic bulk polarizability α_bulk_, computed at the CAM-B3LYP/6-311++G** level, of the selected solvent. Note in fact that in the FQ formalism, η and α are strictly related.^58^

Note that, although the aforementioned validation criteria could have been included in the loss function, we preferred to keep the loss function independent of model specific features. It is worth remarking that the procedure may provide different optimal parameter sets, also in case the constraints mentioned above are incorporated in the loss function.

#### 2.1.5 Optimal Parameter Set

The optimal parameter set, that is, that associated with the lowest value of the loss function and the lowest error for both α_mol_ and α_bulk_, was selected. The values obtained for each solvent molecule are reported in Table S1 given in the Supporting Information.

In Figure 4, FQ solvent–dipole interaction energies as a function of the solvent–dipole distance are reported. Reference QM/dipole values are also plotted for the sake of comparison. The FQ optimal parameter sets correctly reproduce reference curves, although with some discrepancies, essentially due to the lack of out-of-plane polarization in the FQ model (see, for instance, *d*_4_ and *d*_5_ in Figure 4d). This limitation of FQ may be overcome by adding terms depending on atomic dipoles, as it has been recently shown by some of us.^35^ Finally notice that our procedure permits to select the best parameters to reproduce the most relevant interactions of a specific solvent (see for instance *d*_3_–*d*_4_ vs *d*_1_–*d*_2_ in Figure 4c).

### 2.2 Computational Protocol

In order to compute the QM/FQ UV–vis absorption spectra of the selected dyes in the aforementioned solvents, dynamical aspects of the solvation phenomenon were considered by resorting to classical MD simulations. PNA, QB, and MER geometries were optimized at the CAM-B3LYP/aug-cc-pVDZ level, whereas we adopted the CAM-B3LYP/6-31+G(d,p) level for BET, according to ref (38). Solvent effects on molecular geometries were modeled by using the PCM.^11^ All MD runs were performed by using GROMACS.^59^ The general AMBER force field (GAFF) was exploited to describe both intramolecular and intermolecular interactions.^60,61^ Solute- and solvent-bonded and non-bonded parameters were generated by means of the Antechamber package^62,63^ with the only exception of WTR for which the standard TIP3P force field was used.^64^ Atomic charges of both the solute and solvent molecules were calculated by using the RESP charge-fitting method.^65^ During each MD run, solutes were constrained in their minimum energy structure.

The size of the simulation boxes ranged from 7 nm (PNA) to approximately 10 nm (BET). An integration time step of 1 fs was adopted for all MD runs. The temperature was kept constant to 300 K by adopting the velocity-rescaling method^66^ with a coupling constant of 0.1 ps. Electrostatic interactions were taken into account by means of the particle mesh Ewald method^67^ using a cut-off radius of 1.4 nm in real space. The same cutoff was also used for van der Waals interactions.

An NPT simulation of 1 ns (using the Berendsen barostat^68^ and a coupling constant of 2.0 ps) was performed on each system for equilibration purposes. Then, a 2.5 ns *NVT* production run was performed in order to sample the system’s configuration space. A total of 100 uncorrelated snapshots were extracted from the last 2 ns of the MD (one snapshot every 20 ps). For each snapshot, a sphere of variable radius (20–25 Å), depending on the solute size, centered at the solute center of mass, was cut and used in the following QM/FQ UV–vis calculations.

QM/PCM and QM/FQ vertical excitation energies were calculated at the TD-DFT level by exploiting the linear response (LR) and corrected LR (cLR) regimes, which is a first-order state-specific approximation.^36,46^ For all the transitions, both LR and cLR shifts with respect to the frozen density approximation (ω_0_) were summed, in agreement to refs (69) and (70). Such an approach has been recently proposed in the literature and named cLR^2^.^71^ All QM/PCM and QM/FQ ω_0_, LR, cLR, and cLR^2^ energies are reported in Tables S2 and S3 in the Supporting Information. All TD-DFT calculations were performed by using the CAM-B3LYP functional in combination with the aug-cc-pVDZ as basis set (PNA, QB, and MER) or 6-31+G(d,p) (BET), the latter according to ref (38). All QM/FQ calculations were performed by using a locally modified version of the Gaussian 16 package.^72^ Non-polarizable QM/electrostatic embedding (QM/EE) was also performed by exploiting TIP3P and GAFF charges, for WTR and the other solvents, respectively (see Table S4 in the Supporting Information for QM/EE vertical excitation energies).

## 3 Numerical Results

In this section, we apply the parameters obtained above to describe the absorption spectra and solvatochromic shifts of the dyes in Figure 1. For each molecule, we analyze the transition involved in the spectral signal, and we compare the QM/FQ values with EE (QM/EE) and continuum solvation approaches (QM/PCM), so as to disentangle the role of explicit solute–solvent interactions (when relevant) and polarization effects. Finally, the computed data are compared with the experimental values (see Tables S5 in the Supporting Information) and the general trends are commented.

### 3.1 *para*-Nitroaniline

**Figure 5:**
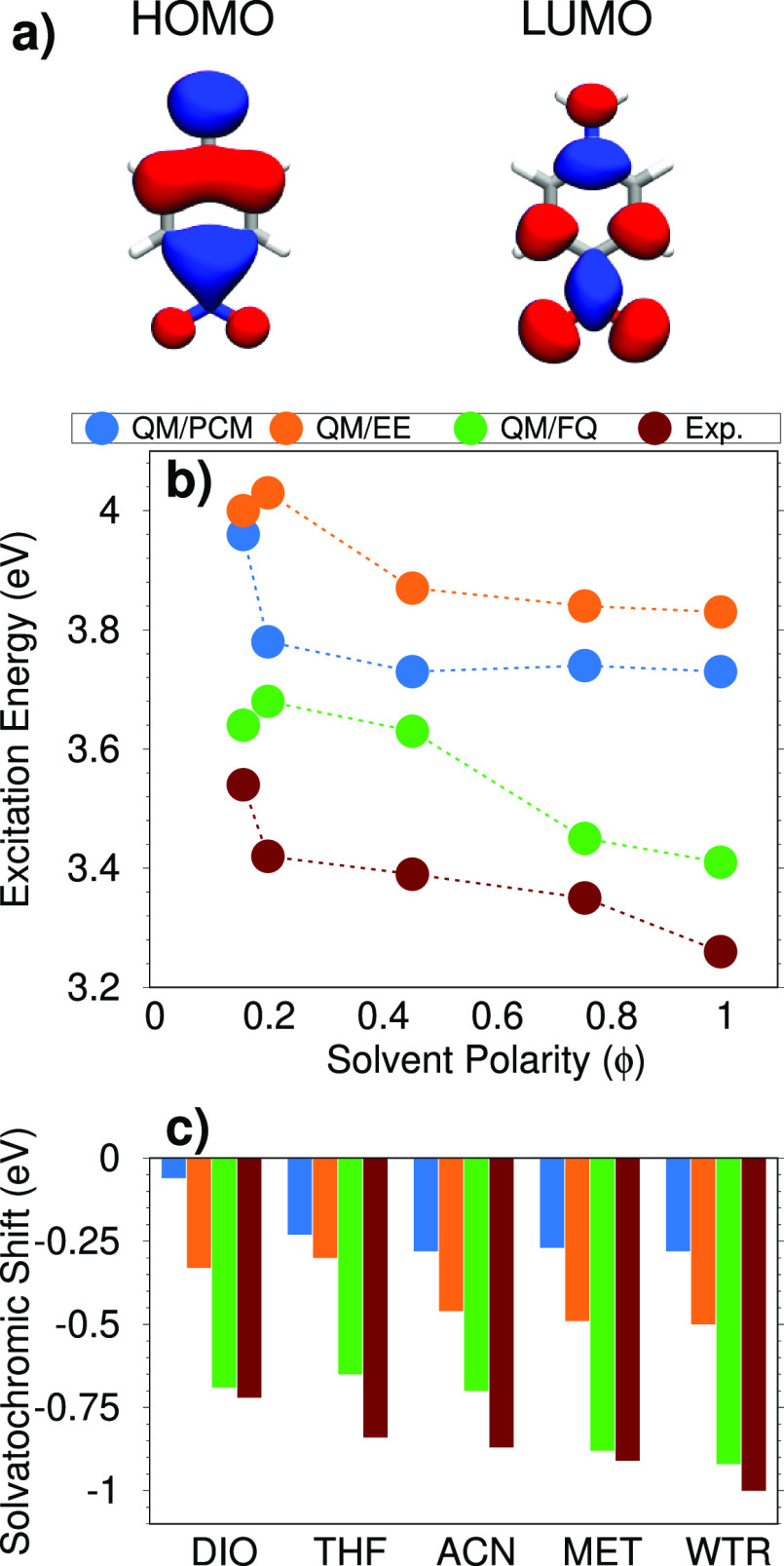
(a) PNA HOMO and LUMO involved in the studied electronic transition. (b) QM/PCM, QM/EE, QM/FQ, and experimental PNA excitation energies as a function of the solvent polarity (ϕ). (c) QM/PCM, QM/EE, QM/FQ, and experimental PNA solvatochromic shifts computed with respect to gas-phase excitation energy.

PNA belongs to the family of “push–pull” organic compounds, being characterized by an electron-donor amino group (NH_2_) and a para electron-acceptor nitro group (NO_2_), which are connected by a π-conjugated phenyl ring (see Figure 1a). Different theoretical approaches (continuum and atomistic) have already been challenged to reproduce PNA solvatochromic shifts, by also exploiting correlated wavefunctions.^73−75^ The absorption spectrum of PNA is characterized by a bright band, which is due to a charge transfer (CT) transition from the donor to the acceptor moieties.^73−80^ Similar to most CT transitions, the PNA absorption maximum exhibits a large red solvatochromic shift as the polarity of the solvent increases. Therefore, PNA represents an ideal candidate to test the quality of the FQ parametrization discussed above. Here, we discuss the PNA absorption spectra in DIO, THF, ACN, MET, and WTR. For all methods (QM/FQ, QM/EE, and QM/PCM), the lowest bright excitation is predicted to be the highest occupied molecular orbital (HOMO)–lowest unoccupied molecular orbital (LUMO) π → π* transition, with a clear CT character (see Figure 5a).

In Figure 5b, QM/FQ, QM/EE, and QM/PCM excitation energies are compared as a function of the solvent polarity (ϕ). We first notice that, as expected, QM/PCM absorption energies remain almost constant when ϕ is larger than 0.4. This is not surprising, due to the well-known asymptotic behavior of PCM contributions for solvents’ permittivity constants larger than 20.^11^ Similar results are given by QM/EE, whereas QM/FQ excitation energies decrease when ϕ increases, thus matching the experimental trends (see 5).^76,79^ For THF and DIO, the experimental trend is generally better reproduced by QM/PCM as compared to QM/FQ. This may be due to inaccurate calculation of the QM/FQ excitation energy of PNA dissolved in THF, because for THF the same FQ atomic parameters obtained for DIO are used (see above).

In Figure 5c, QM/FQ, QM/EE, and QM/PCM solvatochromic shifts (Δ*E*) computed as
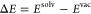
2are compared with the experimental values. Notice that eq 2 refers to vacuo-to-solvent solvatochromic shifts, defined according to ref (38). In eq 2, *E*^vac^ and *E*^solv^ are excitation energies in vacuo and in solution, respectively. Figure 5c clearly shows that both QM/PCM and QM/EE strongly underestimate experimentally measured shifts, whereas QM/FQ gives very good values, in some cases in perfect agreement with experiments. In more detail, QM/FQ gives errors for VAC → DIO, VAC → THF, VAC → ACN, VAC → MET, and VAC → WTR shifts of 0.03, 0.19, 0.17, 0.03, and 0.08 eV, respectively, thus confirming the reliability of both the method and its parametrization for the different solvating environments. As expected, the largest error occurs for VAC → THF, because, as already commented, for THF the same parameters as DIO are exploited.

It is worth noticing that our simulations disregard solute–solvent Pauli repulsion, dispersion effects, dynamical changes in solute configurations, and vibronic effects; therefore, a perfect agreement with experimental values is not expected.^81,82^ However, the exploited computational protocol permits us to disentangle electrostatic/polarization effects on the solute response. The reduced error obtained by exploiting QM/EE and QM/FQ approaches as compared to the implicit (QM/PCM) method shows that an accurate, dynamic description of the solute–solvent interactions is needed, for both apolar (DIO) and polar solvents. Also, the polarizable QM/FQ outperforms the non-polarizable QM/EE when compared to the experimental data, thus showing that solute–solvent polarization plays a crucial role in determining the electronic properties of PNA in solution. Finally, the almost perfect agreement between QM/FQ and the experimental data clearly demonstrates the reliability of the novel FQ parametrization.

### 3.2 Quinolinium Betaine

Quinolinium betaine (QB, see Figure 1) has a zwitterionic character, therefore it is strongly hydrophilic. Its absorption spectrum is dominated by the transition from the dipolar ground state (GS) to an excited state (ES) of considerably reduced polarity.^83^ The GS is, therefore, stabilized in polar solvents, and this leads to an increase of the transition energy, that is, to a positive solvatochromism. In this work, we have investigated the solvatochromic shift of QB dissolved in DIO, THF, ACN, MET, and WTR.

**Figure 6:**
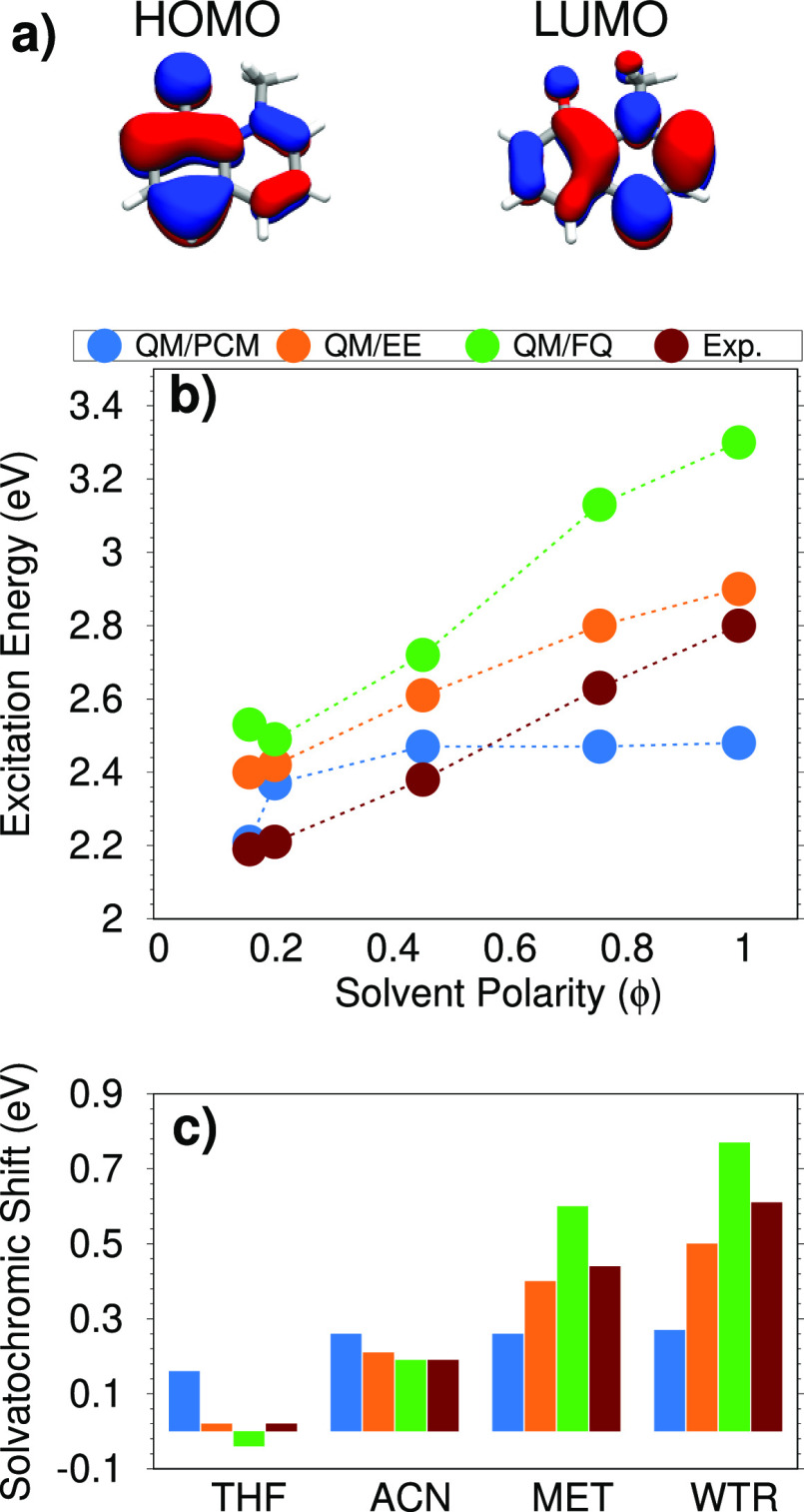
(a) QB HOMO and LUMO involved in the studied electronic transition. (b) QM/PCM, QM/EE, QM/FQ, and experimental QB excitation energies as a function of the solvent polarity (ϕ). (c) QM/PCM, QM/EE, QM/FQ, and experimental QB solvatochromic shifts computed with respect to the excitation energy in dioxane.

For all solvents and embedding methods, the lowest bright excitation of QB corresponds to the HOMO–LUMO π → π* transition, which has a partial CT character (see Figure 6a). Calculated QM/PCM, QM/EE, and QM/FQ excitation energies as a function of solvent polarity (ϕ) are reported in Figure 6b, together with the experimental values taken from ref (83). Similar to PNA, QM/PCM is able to reproduce the experimental trend only for the less-polar solvents (DIO and THF), while, as expected, the curve is substantially flat for the most polar solvents (ACN, MET, and WTR). On the contrary, both QM/EE and QM/FQ well reproduce the experimental trend. Different from PNA, in this case, QM/EE excitation energies are closer to experiments than QM/FQ ones. Such a good agreement may be ascribed to systematic errors due to the selected level of theory;^38^ therefore, in the following we will mainly focus on energy differences, that is, solvatochromic shifts, which are less affected by systematic errors.

Finally, in Figure 6c, computed and experimental solvatochromic shifts are reported. Note that they are calculated with respect to DIO, due to the lack of experimental spectra of QB in the gas phase reported in literature. Figure 6c clearly shows that QM/PCM cannot reproduce the experimental trend for the most polar solvents, whereas both QM/EE and QM/FQ computed solvatochromic shifts increase as the solvent polarity increases. In particular, we notice that for ACN, the two atomistic approaches yield almost the same value (with QM/FQ being in almost perfect agreement with experiments). For MET and WTR, QM/FQ computed shifts are larger than their experimental counterparts, whereas the opposite occurs for QM/EE. This behavior can be due to the fact that Pauli repulsion effects, which might be large for MET and, especially, WTR,^81^ are neglected in our calculations. The inclusion of such effects, which are always repulsive, would bring computed values toward the experimental findings because they act in the opposite direction with respect to QM/FQ electrostatic and polarization contributions.

To conclude this section on QB, it is worth noticing that the QM/FQ solvatochromic shift in THF is wrongly predicted in sign (negative instead of positive). However, the QM/FQ error is of about 0.06 eV (∼1.3 kcal/mol), thus the absolute discrepancy can be considered satisfactory.

### 3.3 Brooker’s Merocyanine

MER also known as Brooker’s merocyanine^84^ has been amply studied both experimentally and theoretically due to the sensitivity of its absorption spectrum on the solvent polarity.^85−88^ Similar to PNA and QB, MER is characterized by two resonance structures, neutral (quinoid) and zwitterionic (benzenoid). The latter is stabilized by polar solvents, whereas the neutral form is predominant in apolar solvents. Also, the phenolate oxygen atom can form hydrogen bonds with protic solvents, thus further increasing the weight of the zwitterionic form.^88−90^ For the aforementioned reasons, MER is used as an indicator to measure solvents’ polarity and hydrogen bond donor capability.

**Figure 7:**
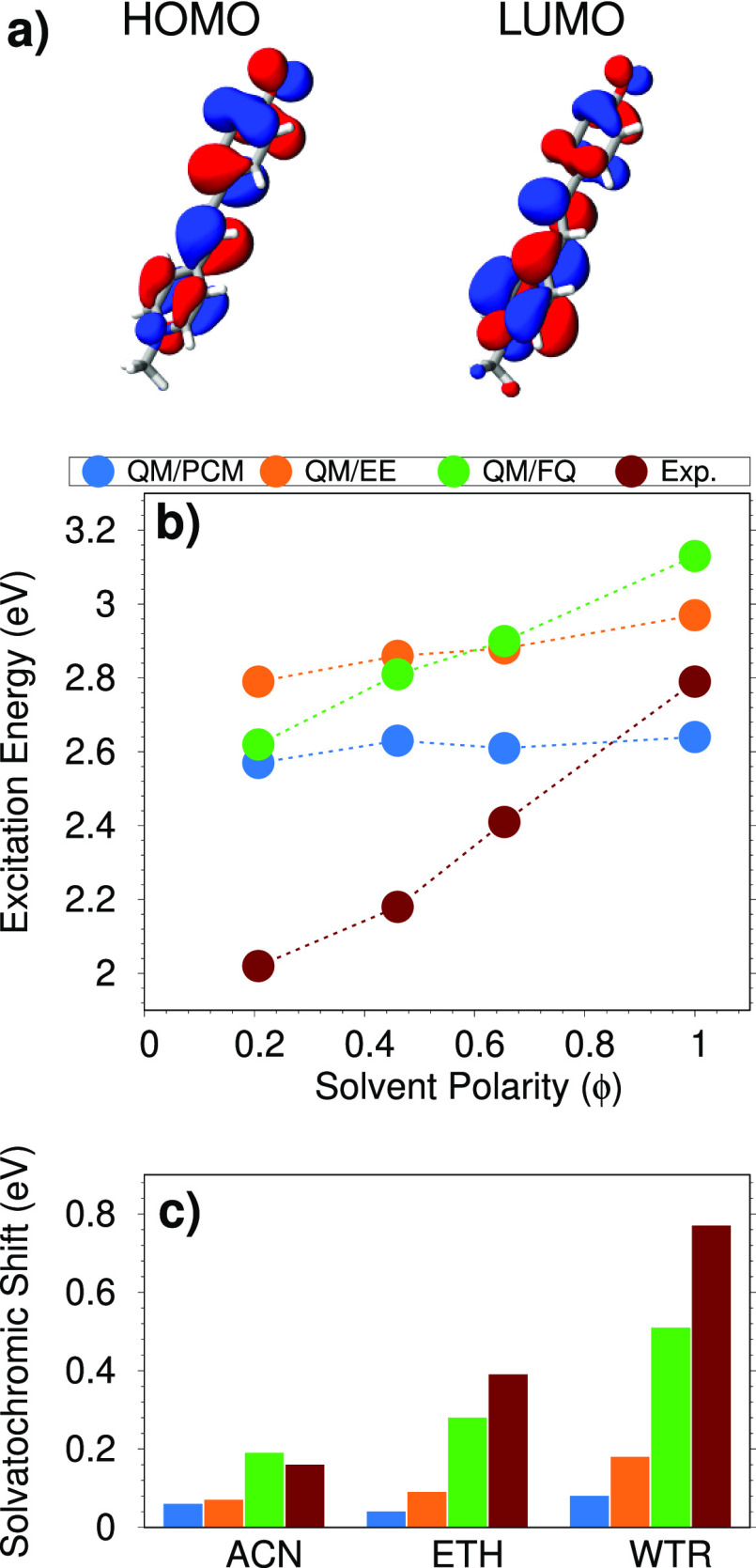
(a) MER HOMO and LUMO involved in the studied electronic transition. (b) QM/PCM, QM/EE, QM/FQ, and experimental MER excitation energies as a function of the solvent polarity (ϕ). (c) QM/PCM, QM/EE, QM/FQ, and experimental MER solvatochromic shifts computed with respect to excitation energy in dioxane.

In this work, we focus on the MER absorption spectra in THF, ACN, ETH, and WTR. MER lowest bright excitation corresponds to a HOMO–LUMO π → π* transition, independent of the solvent and the solvation approach. Figure 7 reports the pictures of MOs involved in the transition, which clearly has CT character, as the density moves from nitrogen to oxygen. In Figure 7b, experimental^86,87^ MER excitation energies as a function of the solvent polarity are compared with the computed results obtained at the QM/PCM, QM/EE, and QM/FQ levels. QM/PCM excitation energies are placed around 2.6 eV, in sharp contrast with experiments, which report an almost linear increase of transition energies by increasing the solvent polarity. Remarkably, this trend is reproduced by QM/FQ, whereas, similar to QM/PCM, QM/EE excitation energies are almost constant in all solvents, with a slight increase when MER is dissolved in WTR.

Computed and experimental solvatochromic shifts with respect to DIO are reported in Figure 7c. Although QM/FQ solvatochromic shifts are underestimated for ETH and WTR, the QM/FQ results directly follow the experimental trend, whereas both the QM/PCM and QM/EE solvatochromic shifts are almost unaffected by solvent polarity. Such a behavior demonstrates the important role of both the specific and polarization solute–solvent effects. To conclude the discussion on MER, we also computed MER gas-phase vertical excitation energy, which is reported in Tables S4 and S6, given as Supporting Information. QM/FQ is able to reproduce both the experimentally measured positive and negative solvatochromic shifts in highly polar (ETH and WTR) and medium-to-low polarity solvents, respectively;^89^ remarkably, such a behavior cannot be described using QM/PCM.

### 3.4 Reichardt’s Betaine (BET)

Solvent effects induce dramatic shifts in the BET absorption spectrum, and for this reason BET has been used to develop the Reichardt’s polarity index ET(30), which is probably the most popular solvent polarity scale.^2,9,91,92^

**Figure 8:**
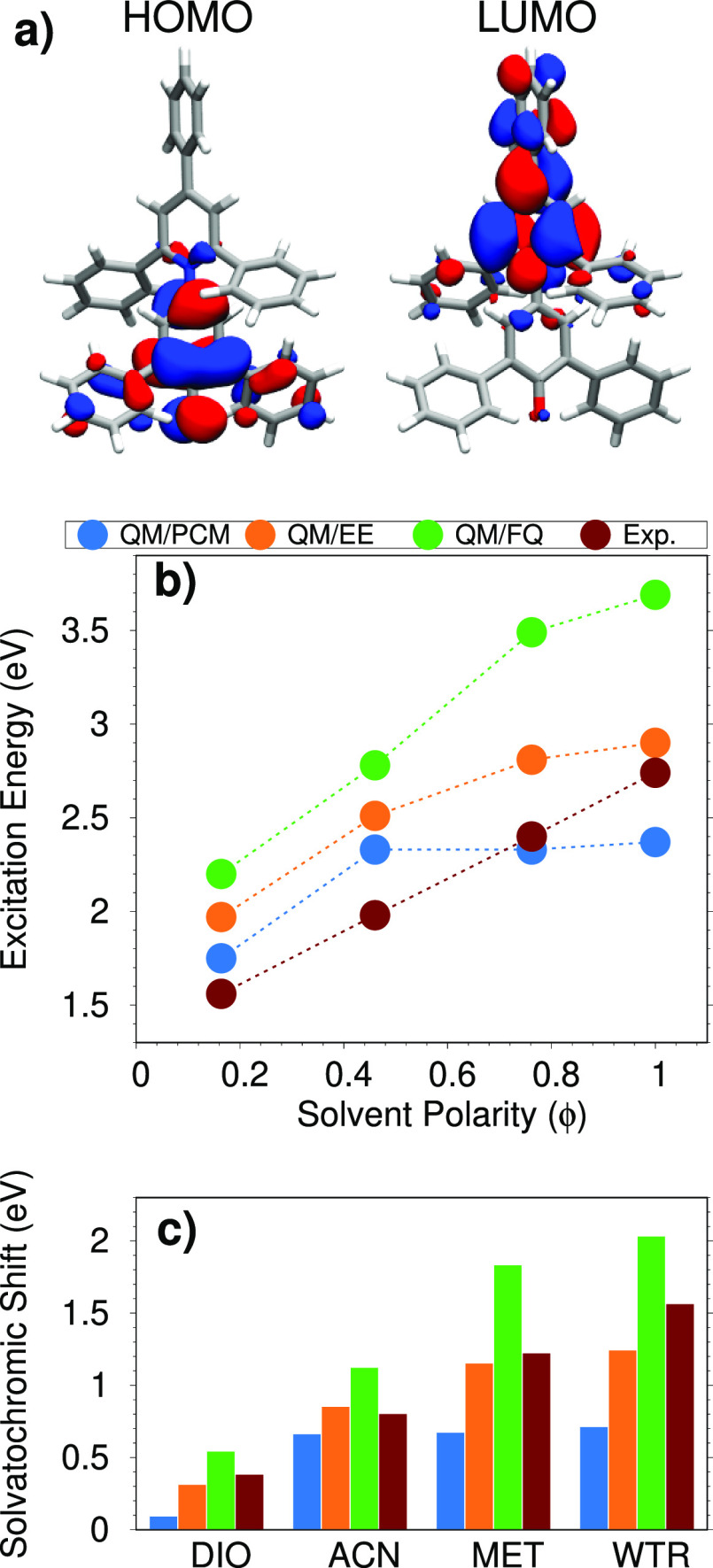
(a) BET HOMO and LUMO involved in the studied electronic transition. (b) QM/PCM, QM/EE, QM/FQ, and experimental BET excitation energies as a function of the solvent polarity (ϕ). (c) QM/PCM, QM/EE, QM/FQ, and experimental BET solvatochromic shifts computed with respect to gas-phase excitation energy.

The absorption spectrum of BET as dissolved in DIO, ACN, MET, and WTR has been computed by modeling the solvent effects by means of the QM/PCM, QM/EE, or QM/FQ approaches. BET lowest excitation, of clear CT character (see 8a), corresponds to the HOMO–LUMO π → π* transition for all considered solvents and solvation approaches. Experimental^86,87^ excitation energies as a function of the solvent polarity are given in Figure 8b, together with the computed values. Clearly, the experimental data linearly depend on the solvent polarity, whereas both the QM/EE and QM/PCM values flatten out by increasing the solvent polarity. Remarkably, among the tested approaches, QM/FQ is the only one that is able to model the experimentally observed trend.

In Figure 8c, we finally report computed and experimental solvatochromic shifts. Reference excitation energies in vacuo are 1.66 (experimental) and 1.18 (calculated) eV. BET values confirm what has already been discussed for the previous molecules. QM/PCM cannot reproduce experimental solvatochromic shifts for the most polar and protic solvents, whereas the QM/EE results are in good agreement with the experimental results, especially for DIO, ACN and MET. QM/FQ always overestimates the experimental values, similar to the case of QB (see above). However, also for BET the good performance of QM/EE can be ascribed to a fortunate error cancellation, due to the fact that both Pauli repulsion and solute–solvent polarization effects are neglected (the latter are instead considered by QM/FQ, and are large).

### 3.5 Discussion

**Figure 9:**
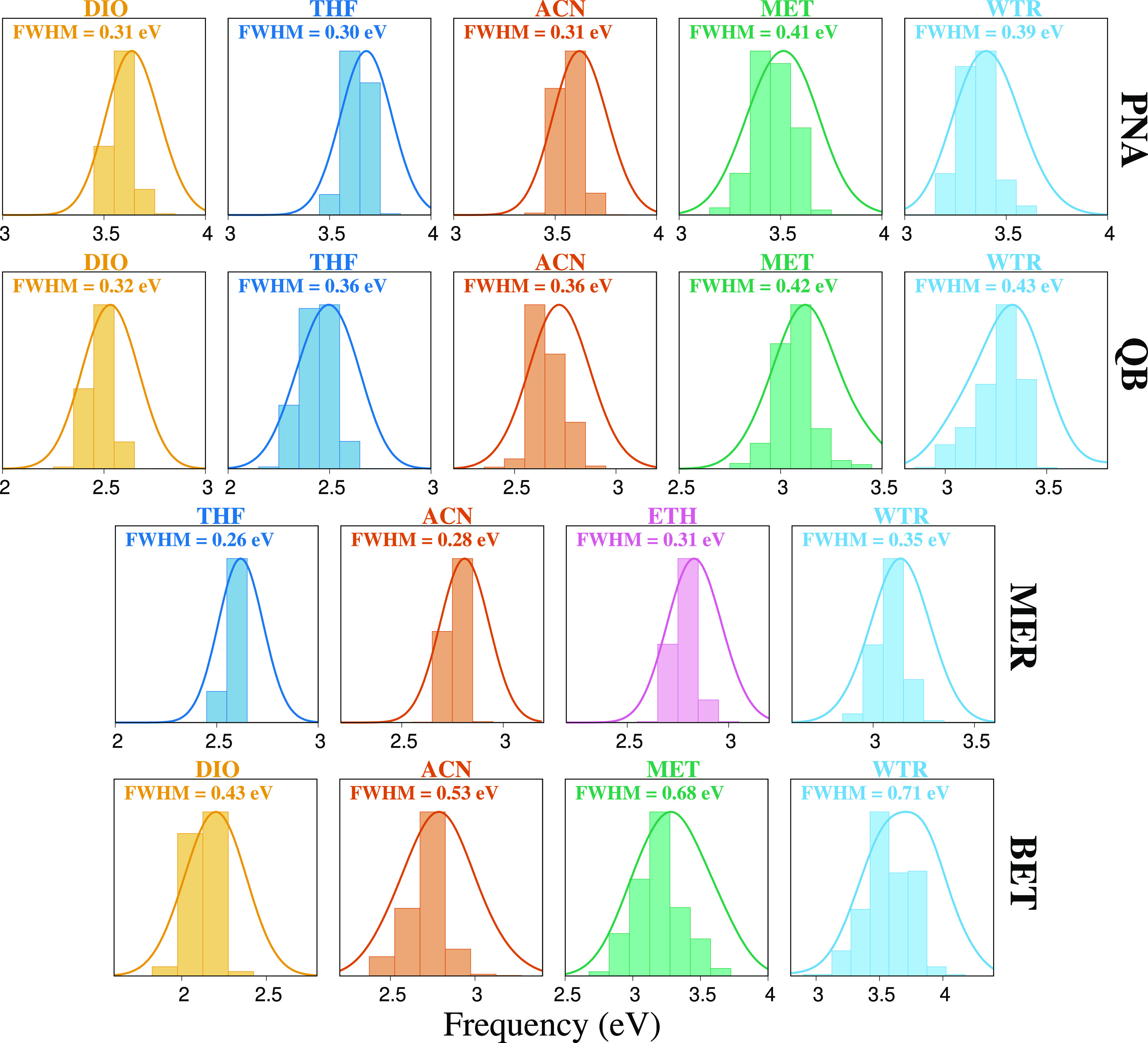
QM/FQ excitation energy distributions of PNA, QB, MER, and BET dissolved in different solvents of increasing polarity. The fwhm of each band is also reported (in eV).

In this section, we further study the quality of the computational approach, by focusing on general trends for all four molecules. First, we investigate band broadening by moving from apolar to polar protic solvents. To this end, the stick spectra, displayed as histograms, of the four chromophores in the different solvents are reported in Figure 9. Clearly, by moving from DIO to WTR, the absorption band broadens out. By fitting the different absorption spectra with Gaussian functions, we see that their full width at half-maximum (fwhm) values, which are a direct measure of band broadening, are substantially influenced by the solvent. The increase of band broadening is particularly evident for BET, for which the fwhm in WTR is 65% larger than that in DIO (0.71 vs 0.43 eV). Because BET is kept frozen during MD runs, broadening arises from fluctuations of solvent molecules around the solute. From the physicochemical point of view, this is not unexpected. In fact, for strongly interacting solvents, such as MET and WTR, which can interact with BET via HB interactions, such fluctuations yield to a larger scattering of absorption energies as compared to apolar non-protic solvents, such as DIO.

**Figure 10:**
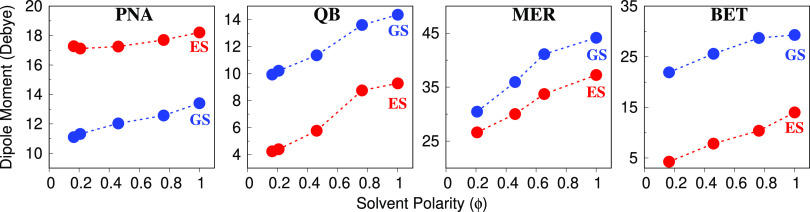
QM/FQ PNA, QB, MER, and BET ground (GS, blue) and excited (ES, red) dipole moments (in debye) as a function of the solvent polarity index, (ϕ).

The reliability of the method can also be investigated by studying the dependence of GS and ES dipole moments (in debye) as a function of the solvent polarity ϕ (see Figure 10). We first notice that PNA differs from other solutes, because its GS dipole moment is lower than the ES one, independently of the solvent polarity. This is clearly reflected by the negative solvatochromic shifts reported for PNA only. However, for all studied systems, an almost linear dependence of both GS and ES dipole moments as a function of the solvent polarity is predicted. Remarkably, ETH, MET, and WTR values (see for instance MER, QB, and BET) deviate from linearity, being such a behaviour possibly explained by the fact that protic solvents may not be barely described by the solvent polarity index, which does not take into account strong, directional solute–solvent interactions.

**Figure 11:**
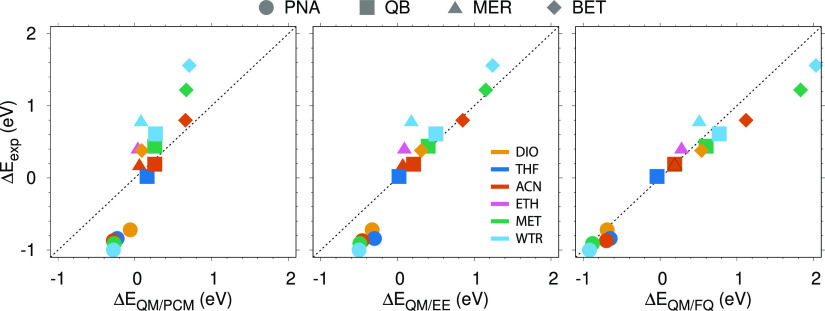
PNA, QB, MER and BET QM/PCM (left), QM/EE (middle), and QM/FQ (right) solvatochromic shifts (Δ*E*) with respect to the experimental values (Δ*E*_exp_).

Finally, in Figure 11, correlation maps between the experimental (Δ*E*_exp_) solvatochromic shifts and the calculated QM/PCM (left panel, Δ*E*_QM/PCM_), QM/EE (middle panel, Δ*E*_QM/EE_), and QM/FQ (right panel, Δ*E*_QM/FQ_) values are reported.

For PNA (circles), QM/FQ deviations with respect to experimental values oscillate from a minimum value of 0.03 eV for DIO and MET to a maximum value of 0.19 eV for THF. A similar behavior is observed for QB, where the largest discrepancy is observed for WTR (0.17 eV), whereas for MER, the largest error increases up to 0.26 eV (WTR). As already commented above, the largest deviations are observed for BET. However, we remark that such discrepancies can be attributed to the solute geometry being kept frozen during MD runs and also to the fact that solute–solvent interactions are limited to electrostatic (and polarization) contributions, and other quantum forces may be in place for some of the studied molecules. This is for instance the case of BET in WTR; in fact, if Pauli repulsion is included by resorting to the approach that we have presented in ref (23) and extended to TD-DFT in ref (81), for aqueous BET the error moves from 0.46 to 0.1 eV. We finally remark that vibronic effects may affect computed accurate solvatochromic shifts.^82^ Gas-phase vibronic UV–vis spectra of the studied systems within the vertical gradient approximation^93^ are reported in Table S6 in the Supporting Information, showing that such terms can be as large as 0.1 eV.

Finally, let us compare QM/FQ to QM/EE and QM/PCM. QM/FQ outperforms the other two approaches, resulting in mean error of about 0.18 eV with respect to 0.25 and 0.43 eV, as it is obtained by using QM/EE and QM/PCM, respectively. Not surprisingly, QM/PCM gives the worst values, thus showing the limitations of continuum solvation and the necessity of explicit treatment of protic, polar solvents. On the other hand, the reduced errors of QM/FQ compared to those of QM/EE demonstrate that polarization effects are relevant to the description of solvatochromic shifts.

## 4 Summary and Conclusions

In this work, we have extended the applicability of QM/FQ to different solvents of various polarities and hydrogen-bonding capabilities. QM/FQ has been challenged to reproduce solvatochromic shifts of four dyes dissolved in different solvents, giving a reliable description of the experimental trends. Remarkably, in most cases, the QM/FQ results are in much better agreement with the experimental values than other approaches, which lack the description of polarization effects (QM/EE) or any atomistic description of the solvent molecules (QM/PCM), the latter being particularly relevant for protic solvents. Also, QM/FQ errors with respect to the experiments are larger when non-electrostatic interactions play a crucial role. Notably, neither THF nor ETH were specifically parameterized by means of the proposed parametrization procedure. In fact, in both cases, we exploited the optimized parameters for DIO and MET, respectively. This demonstrates a good level of transferability of the parameters to treat molecules with similar chemical structures.

The FQ force field lacks a multipolar expansion of electrostatic interactions, because it only uses electric charges; therefore, short-range electrostatics might be wrongly described. Such an approximation seems not to be particularly relevant for the studied cases; however, it can be solved by extending the electrostatic expansion, as it has been pragmatically proposed by some of us for the QM/FQFμ approach,^35^ which can be parametrized for various solvents in a similar way to that studied in this work. This topic is currently under investigation in our group, and will be the topic of a forthcoming communication. Finally, geometrical effects are crucial to get a good reproduction of experimental excitation energies. Therefore, the QM/FQ results can be further improved by resorting to QM/MM simulations, possibly coupled to enhanced sampling techniques.^94^ Investigation of these issues is underway and will be reported in future works.

## Supporting Information Available

FQ parameters for the selected solvents and QM/FQ, QM/PCM, QM/EE, and experimental vertical excitation energies (PDF)
