# Supplementary material for: Quantum Mechanics/Fluctuating Charge Protocol to Compute Solvatochromic Shifts
Source: J Chem Theory Comput. 2021 Oct 7;17(11):7146–56. doi: 10.1021/acs.jctc.1c00763 (PMC8582258; doi:10.1021/acs.jctc.1c00763)
Supplement: Supplementary file 1 — ct1c00763_si_001.pdf [file ct1c00763_si_001.pdf]

Supporting Information - QM/Fluctuating Charge Protocol to Compute Solvatochromic Shifts

Matteo Ambrosetti, Sulejman Skoko, Tommaso Giovannini,* and Chiara Cappelli*

Scuola Normale Superiore, Piazza dei Cavalieri 7, 56126 Pisa, Italy.

E-mail: tommaso.giovannini@sns.it; chiara.cappelli@sns.it

Table S1: FQ optimal parameters (electronegativity χ and chemical hardness η) obtained by exploiting the parametrization procedure. All atomic electronegativities are reported as differences with respect to the least electronegative atom, i.e. Hydrogen ($\Delta\chi_{X-H}$). All data are given in a.u.

Solvent	$\Delta\chi_{C-H}$	$\Delta\chi_{N-H}$	$\Delta\chi_{O-H}$	η_H	η_C	η_N	η_O
DIO	0.09	–	0.14	0.58	0.40	–	0.46
ACN	0.04	0.16	–	0.57	0.38	0.45	–
MET	0.18	–	0.37	0.52	0.17	–	0.95
WTR	–	–	0.15	0.44	–	–	0.54

Table S2: QM/PCM ω_0, LR, cLR and cLR^2 excitation energies of PNA, QB, MER and BET dissolved in the selected solvents. All data are reported in eV.

**PNA**				
Solvent	ω_0	LR	cLR	cLR^2
DIO	4.15	4.04	4.07	3.96
THF	4.00	3.88	3.90	3.78
ACN	3.93	3.82	3.84	3.73
MET	3.93	3.83	3.85	3.74
WTR	3.92	3.81	3.84	3.73
**QB**				
Solvent	ω_0	LR	cLR	cLR^2
DIO	2.32	2.30	2.23	2.21
THF	2.51	2.49	2.39	2.37
ACN	2.61	2.59	2.48	2.47
MET	2.61	2.59	2.49	2.47
WTR	2.62	2.61	2.50	2.48
**MER**				
Solvent	ω_0	LR	cLR	cLR^2
THF	2.82	2.58	2.80	2.57
ACN	2.86	2.66	2.82	2.63
ETH	2.85	2.65	2.81	2.61
WTR	2.87	2.68	2.83	2.64
**BET**				
Solvent	ω_0	LR	cLR	cLR^2
DIO	1.94	1.92	1.78	1.75
ACN	2.60	2.58	2.34	2.33
MET	2.59	2.58	2.34	2.33
WTR	2.64	2.63	2.38	2.37

Table S3: QM/FQ ω_0, LR, cLR and cLR^2 excitation energies of PNA, QB, MER and BET dissolved in the selected solvents. All data are reported in eV.

**PNA**				
Solvent	ω_0	LR	cLR	cLR^2
DIO	3.86	3.72	3.78	3.64
THF	3.86	3.74	3.80	3.68
ACN	3.77	3.67	3.73	3.63
MET	3.70	3.56	3.65	3.45
WTR	3.61	3.46	3.57	3.41
**QB**				
Solvent	ω_0	LR	cLR	cLR^2
DIO	2.64	2.62	2.55	2.53
THF	2.59	2.57	2.51	2.49
ACN	2.80	2.78	2.74	2.72
MET	3.30	3.26	3.17	3.13
WTR	3.46	3.41	3.35	3.30
**MER**				
Solvent	ω_0	LR	cLR	cLR^2
THF	2.87	2.63	2.85	2.62
ACN	3.00	2.83	2.98	2.81
ETH	3.26	2.98	3.19	2.90
WTR	3.38	3.18	3.33	3.13
**BET**				
Solvent	ω_0	LR	cLR	cLR^2
DIO	2.45	2.43	2.22	2.20
ACN	2.95	2.94	2.79	2.78
MET	3.57	3.55	3.30	3.49
WTR	3.90	3.88	3.71	3.69

Table S4: QM/EE Excitation energies (eV) of the studied molecules dissolved in the selected solvents. Computed excitation energies in gas-phase (VAC) are also given.

Solute	VAC	DIO	THF	ACN	ETH	MET	WTR
PNA	4.33	4.00	4.03	3.87	–	3.84	3.83
QB	2.14	2.40	2.42	2.61	–	2.80	2.90
MER	2.92	–	2.79	2.86	2.88	–	2.97
BET	1.66	1.97	–	2.51	–	2.81	2.90

Table S5: Experimental excitation energies (eV) of the studied molecules dissolved in the selected solvents. Experimental excitation energies in gas-phase (VAC) are also given.

Solute	VAC	DIO	THF	ACN	ETH	MET	WTR
PNA[1,2]	4.26	3.54	3.42	3.39	–	3.35	3.26
QB[3]	–	2.19	2.21	2.38	–	2.63	2.80
MER[4,5]	–	–	2.02	2.18	2.41	–	2.79
BET[6]	1.18	1.56	–	1.98	–	2.40	2.74

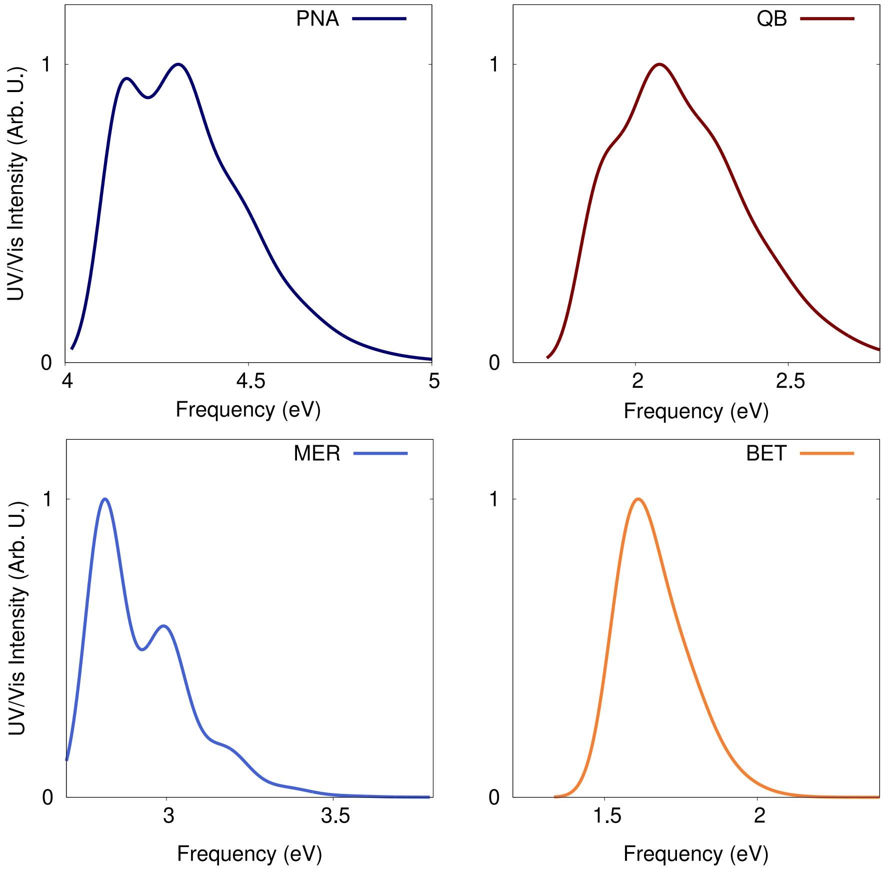

Figure S1: Gas-phase PNA, QB, MER and BET Vibronic Vertical Gradient (VG) UV/Vis Intensities.

Table S6: Gas-phase PNA, QB, MER and BET Vertical and Vibronic Excitation energies maxima. All data are given in eV.

Solute	Vertical	Vibronic (VG)
PNA	4.33	4.31
QB	2.14	2.08
MER	2.92	2.82
BET	1.66	1.61

References

(1) Kovalenko, S.; Schanz, R.; Farztdinov, V.; Hennig, H.; Ernsting, N. Femtosecond relaxation of photoexcited para-nitroaniline: solvation, charge transfer, internal conversion and cooling. *Chem. Phys. Lett.* **2000**, *323*, 312–322.

(2) Stähelin, M.; Burland, D.; Rice, J. Solvent dependence of the second order hyperpolarizability in p-nitroaniline. *Chem. Phys. Lett.* **1992**, *191*, 245–250.

(3) Novaki, L. P.; El Seoud, O. A. Solvatochromism in pure solvents: Effects of the molecular structure of the probe. *Berichte der Bunsengesellschaft für physikalische Chemie* **1996**, *100*, 648–655.

(4) Morley, J. O.; Morley, R. M.; Fitton, A. L. Spectroscopic studies on Brooker's merocyanine. *J. Am. Chem. Soc.* **1998**, *120*, 11479–11488.

(5) Cavalli, V.; da Silva, D. C.; Machado, C.; Machado, V. G.; Soldi, V. The fluorosolvatochromism of Brooker's merocyanine in pure and in mixed solvents. *J. Fluoresc.* **2006**, *16*, 77–86.

(6) Reichardt, C. Solvatochromic dyes as solvent polarity indicators. *Chem. Rev.* **1994**, *94*, 2319–2358.
